# Modelling the cost-effectiveness of human milk and breastfeeding in preterm infants in the United Kingdom

**DOI:** 10.1186/s13561-016-0136-0

**Published:** 2016-12-01

**Authors:** James Mahon, Lindsay Claxton, Hannah Wood

**Affiliations:** York Health Economics Consortium, Enterprise House Innovation Way, University of York, Heslington, York, YO10 5NQ UK

**Keywords:** Breastfeeding, Preterm infants, Economic evaluation, Cost-effectiveness

## Abstract

**Objectives:**

To estimate the cost savings and health benefits in the UK NHS that could be achieved if human milk usage in the NICU was increased.

**Methods:**

A systematic review established the disease areas with the strong sources of evidence of the short, medium and long-term benefits of human milk for preterm infants as opposed to the use of formula milk. The analysis assessed the economic impact of reducing rates of necrotising enterocolitis, sepsis, sudden infant death syndrome, leukaemia, otitis media, obesity and neurodevelopmental impairment.

**Results:**

Based on the number of preterm babies born in 2013, if 100% of premature infants being fed mother’s milk could be achieved in the NICU, the total lifetime cost savings to the NHS due to improved health outcomes is estimated to be £46.7 million (£30.1 million in the first year) with a total lifetime QALY gain of 10,594, There would be 238 fewer deaths due to neonatal infections and SIDS, resulting in a reduction of approximately £153.4 million in lifetime productivity. Sensitivity analyses indicated that results were robust to a wide range of inputs.

**Conclusions:**

This analysis established that increasing the use of human milk in NICUs in the UK would lead to cost savings to the NHS. More research is needed on the medium and long term health and economic outcomes associated with breastfeeding preterm infants, and the differences between mother’s own and donor breast milk.

## Background

The benefits of human milk for both preterm and term infants are well established in the medical literature, with evidence demonstrating that human milk can aid development and reduce risks of certain infections [1].

The World Health Organization (WHO) recommends exclusive breastfeeding for at least the first 6 months of life to provide adequate nutrition for infant development [2, 3]. Yet, the most recent Infant Feeding Survey shows that very few mothers in the UK are following these recommendations: in 2010 only 69% of mothers exclusively breastfed at birth, with rates falling to 1% at 6 months [4]. Barriers to breastfeeding in the UK include personal and society biases towards breastfeeding, issues with feeding in public and employment practices [5].

Mothers with preterm infants face further barriers to breastfeeding. For infants that are born preterm (<37 weeks of pregnancy), providing human milk to the infant and sustaining lactation up to and beyond hospital discharge may be challenging [6]. In the UK, the rate of exclusive breastfeeding at discharge for preterm infants has been found to be as low as 29% and for exclusive of mixed breastfeeding as low as 35% [7]. In these circumstances, feeding with donor breastmilk from other lactating mothers has been shown to have benefits over feeding formula milk, such as a lower risk of necrotising enterocolitis (NEC) in the infant due to the presence of active enzymes and anti-infective properties in the breastmilk [8].

In addition to the health benefits, the economic case for breastfeeding term infants has also been addressed. The 2012 UNICEF report on diseases and developmental deficits associated with low breastfeeding rates in the UK estimated that a moderate increase in breastfeeding could lead to fewer hospital admissions and GP consultations from conditions such as asthma, leukemia, coeliac disease, cardiovascular disease and sepsis [9]. Supporting mothers to breastfeed exclusively for the first 4 months could save the NHS over £11 million per year by reducing the incidence and treatment costs for acute infections such as gastrointestinal and lower respiratory tract infections, and acute otitis media in infants [10]. This is by no means an issue specific to the UK, with a recent study estimating that the international impact of not breastfeeding is associated with economic losses of about $302 billion annually, or 0 · 49% of world gross national income [11].

The economic value of increasing human milk feeding for preterm infants is therefore potentially high. For example, recent research indicates that increasing the rate of breastfeeding at discharge in UK neonatal units could save the NHS £6.12 million per year from reduced costs of treating NEC in preterm infants [10]. However, the total economic value of human milk feeding covering all the short and long term benefits (such as the reduction of chronic conditions such as coronary heart disease (CHD) and obesity [12], and neurodevelopmental impairment (NDI) resulting from acute infections [13]) has not been estimated.

The purpose of the research was explicitly not to demonstrate or speculate on the underlying biological reasons as to why human milk confers benefit to preterm infants or to speculate on how rates could be increased. Rather, the aim of the research was to highlight the potential economic benefit that could result from the increase in human milk consumption on preterm infants based upon previously published odds ratios on specific outcomes depending on whether infants were fed formula or human milk. Specifically, the research aimed to show the potential health benefits and cost savings to the NHS in England and Wales as an exemplar of savings in a developed economy.

## Methods

### Literature review

In constructing the economic model, the first step was to conduct a literature search to identify literature reviews that provided evidence of the benefits of human milk and breastfeeding for preterm infants as opposed to the use of formula milk. The question was defined using the PICOS framework (details given in Online Resource 1) focusing on preterm infants and feeding human milk compared with infant formula. Reported short, medium and long-term benefits or outcomes from human milk and/or breastfeeding were eligible for inclusion. There are a large number of existing evidence syntheses which have summarised, analysed and appraised the primary research evidence in this field. Therefore we chose to include literature reviews to inform the model, with systematic reviews taking precedence over pragmatic reviews.

A focused search of the following resources was undertaken: Cochrane Database of Systematic Reviews (CDSR), Database of Abstracts of Reviews of Effects (DARE), Health Technology Assessment Database (HTA Database) all via the Cochrane Library, and MEDLINE and MEDLINE In-Process via Ovid SP. The searches were limited to material published from 2000 to current in order to prioritise the most recent evidence. The search strategy used to search Ovid MEDLINE is presented in Online Resource 1.

To identify any relevant reviews that might have been missed by the database searches, particularly reviews that have not been published as a journal article or book chapter, webpages of relevant organisations were browsed and/or searched, including the World Health Organisation, The Partnership for Maternal, Newborn and Child Health, UNICEF, Save the Children, USAID, Women and Health Alliance International, World Alliance for Breastfeeding Action, and CARE International.

### Economic modelling

The aim of the economic modelling was to estimate the cost savings and health benefits in the NHS in England and Wales that could be achieved if human milk usage and breastfeeding rates in preterm infants in neonatal intensive care units (NICUs) were increased. England and Wales were chosen rather than the whole of the UK due to data availability in those nations and that the population in England and Wales accounts for approximately 90% of the UK population.

In order to estimate the extent of cost savings and health benefits associated with increased human milk usage and breastfeeding rates in the NICU, two scenarios were defined: the base case scenario which reflects the current level of human milk usage and breastfeeding in preterm infants in England and Wales of 35% [7], and a hypothetical scenario which reflects 100% human milk feeding of preterm infants at least until the age of 6 months. It should be recognized that for preterm infants in the neonatal intensive care unit mothers’ own milk often needs to be fortified and in these instances it is still classed as exclusive human milk feeding and breastfeeding if the infant’s intake is not further supplemented with formula.

The analysis assessed outcomes in infants in the NICU. Infants who were exclusively human milk fed at discharge were compared with infants who were never fed human milk (fed with formula milk). After discharge from NICU, the analysis compared outcomes in infants who received some human milk in the NICU (including those who were exclusively and non-exclusively breastfed) with those who received no human milk (formula fed) infants.

Outcomes were assessed at the population level. The number of preterm infants born in 2013 in England and Wales was 51,703, which was obtained from a dataset published by the Office for National Statistics (ONS) [14].

Outcomes included in the model were identified in the literature review described above, and from reviewing previous economic evaluations of the impact of breastfeeding [9, 10]. For each of the disease outcomes included in the model, an odds ratio was used to model the benefit of breastfeeding to represent the degree to which the rate of the disease outcome would be decreased. To note, the paper is explicitly not about what is determining the odds ratio and why breast milk is beneficial to infants; it is a health economics analysis treating human milk as an intervention using the existing evidence of its effectiveness. The outcomes include: sepsis; necrotising enterocolitis (NEC); sudden infant death syndrome (SIDS); acute otitis media (AOM); childhood leukaemia; childhood obesity and the associated impact on developing Type II Diabetes and coronary heart disease (CHD) later in life; and neurodevelopment impairment (NDI) and disability.

The primary economic analysis took the perspective of the NHS. Costs that fall on individuals, households or any other sectors were excluded. Quality of life was measured in quality-adjusted life years [15]. The costs to the society were included in a secondary analysis, whereby the impact on lifetime economic productivity due to averted deaths was assessed.

Each disease outcome was modelled over different time horizons. For NEC and sepsis, the length of stay in neonatal units was considered as the time horizon for costing. For acute conditions that occur in childhood (AOM and SIDS), it was the first year of life. Disability and the chronic conditions that were associated with childhood obesity (Type II Diabetes and CHD) were modelled over the infants’ lifetime. Where the costing time horizon is longer than a year, a rate of 3.5% per year was used to discount the future stream of treatment costs in baseline estimates [16].

**Fig. 1:**
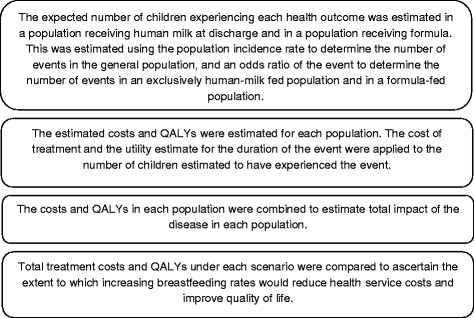
Model diagram. Presents the calculation steps in the model. Abbreviations. QALY: quality-adjusted life year

The model used the following steps to estimate the cost savings and health benefits associated with mothers’ own milk versus formula is outlined in Fig. 1.

A summary of all inputs used in the model is provided in Table 1.

**Table 1:** Modelling inputs

Outcome	Baseline incidence	Odds ratio	Treatment cost	Mortality	Quality of life
Medical NEC	3.5% for birth weight 500–999 g 2.1% for birth weight 1000–1749 g 0.5% for birth weight 1750–2500 g [21]	MM vs MM + formula: 0.412 [22] Formula vs MM and formula: 3.006 [23]	27.2 days stay in NICU [24] Cost per day of £630.08 based on weighted average of neonatal critical care codes [25] Total cost of £1,739	N/A	N/A
Surgical NEC	3.3% for birth weight 500–999 g 0.6% for birth weight 1000–1749 g 0.1% for birth weight 1750–2500 g [21]	As for medical NEC	£1,739 (non-elective inpatient: major neonatal diagnosis) [25] Plus incurs cost of medical NEC treatment. Total cost of £18,877.	N/A	N/A
Sepsis	27.2% for birth weight 500–999 g 8.2% for birth weight 1000–1749 g 4.7% for birth weight 1750–2500 g [26]	MM vs MM + formula: 0.707 [22] Formula vs MM and formula: 0.803 [27]	5.9 days stay in NICU [24] Cost per day of £630.08 based on weighted average of neonatal critical care codes [25] Total cost of £17,138.	N/A	N/A
Mortality in NICU	20.5% for birth weight 500–999 g [28] 8.0% for birth weight 1000–1749 g [29] 5.0% for birth weight 1750–2500 g [30]	If medical NEC: 2.055 [28] If surgical NEC: 3.124 [28] If sepsis. 3.219 [29]	N/A	N/A	Lost (discounted) QALYs per premature death of on average 23.6. Based on mean life expectancy and age-related quality of life [31, 32]
SIDS	0.07% (per live birth) [33]	0.40 (any breastfeeding versus formula) [34]	£72 (VB11Z: Emergency Mediine, No Investigation with No Significant Treatment) [25]	N/A	Lost (discounted) QALYs per premature death of on average 23.6. Based on mean life expectancy and age-related quality of life [31, 32]
AOM	0.14% (for infants under the age of 1) [10]	0.40 (any breastfeeding versus formula) [35]	£46 - visit to a general practitioner [36]	N/A	N/A
Leukaemia	0.04% (cumulative incidence up to age 15) [37]	0.91 (any breastfeeding versus formula) [38]	£114,456 per case of leukaemia (includes all treatment-related costs) [39]	92% survival rate [40]	0.66 on treatment [41]
Obesity	9.5% (proportion children aged 4 to 5 defined as obese) [42] Probability of an obese child being an obese adult: 65% [43]	0.79 (any breastfeeding versus formula) [12]	N/A (see diabetes and CHD)	N/A (see diabetes and CHD)	N/A (see diabetes and CHD)
Type 2 diabetes	9.61% (prevalence in obese adults) [44]	N/A	£787 per year [45]	Age at diagnosis: 55 Life expectancy: 75 [46]	0.866 [45]
CHD	6.04% (prevalence in obese adults) [44]	N/A	£1,974 per year [45]	Age at diagnosis: 65 Life expectancy: 75 (assumption)	0.867 [45]
NDI	49% for birth weight 500–999 g 41% for birth weight 1000–1749 g 34% for birth weight 1750–2500 g [47] Mild NDI: 65% Moderate NDI: 22% Severe NDI: 14% [47]	Given sepsis: 2.282 [29] Given medical NEC: 1.187 [28] Given surgical NEC: 1.985 [28]	Lifetime cost of disability Mild: £14,421 Moderate: £13,959 Severe: £365,005 [48]	Life expectancy (years) Mild: 78.5 Moderate: 67.8 Severe: 26.1 [48]	Mild: 0.85 Moderate: 0.645 Severe: 0.47 [48]

Presents a summary of parameter values used in the economic analysis. Abbreviations: *NEC* necrotizing enterocolitis, *MM* mother’s own milk, *NICU* neonatal intensive care unit, *QALY* quality-adjusted life year, *SIDS* sudden infant death syndrome, *AOM* acute otitis media, *CHD* coronary heart disease, *NDI* neurodevelopmental impairment

## Results

### Results of the literature review

The search strategies retrieved 1,612 records, after deduplication 1,418 records remained, with the majority of records (1,271) sourced from MEDLINE. After an initial screening, 845 records were reviewed for further assessment.

As this is a pragmatic review with the purpose of identifying evidence for benefits of human milk and/or breastfeeding, only the most recent reviews reporting either a specific outcome or at one of our three time points of interest were included. Other reviews were only considered for inclusion if they provided either information from different individual studies or on different outcomes at the same time point. Studies on term infants were only considered where there was an absence of evidence at a given time point or on a specific outcome at a given time point for preterm infants.

### Outcomes and evidence availability

The majority of evidence on outcomes is for term infants, with no reviews providing evidence of long-term outcomes of either expressed human milk or breastfeeding to preterm infants. The only medium-term outcome for preterm infants is NEC and it is not clear in the review at what time point this was relevant. In addition, this was a review of donor human milk rather than the mother’s own milk [17]. The short-term outcomes are from a review of low birth weight rather than preterm infants [13].

The evidence is mixed for long-term outcomes. For some outcomes, such as obesity and IQ, some reviews conclude there is no effect of human milk or breastfeeding, and others conclude that there is.

More details on the availability of evidence on outcomes for preterm and term infants and included reviews can be found in Online Resource 1.

### Results of the economic model

#### Outcomes in the NICU

**Fig. 2:**
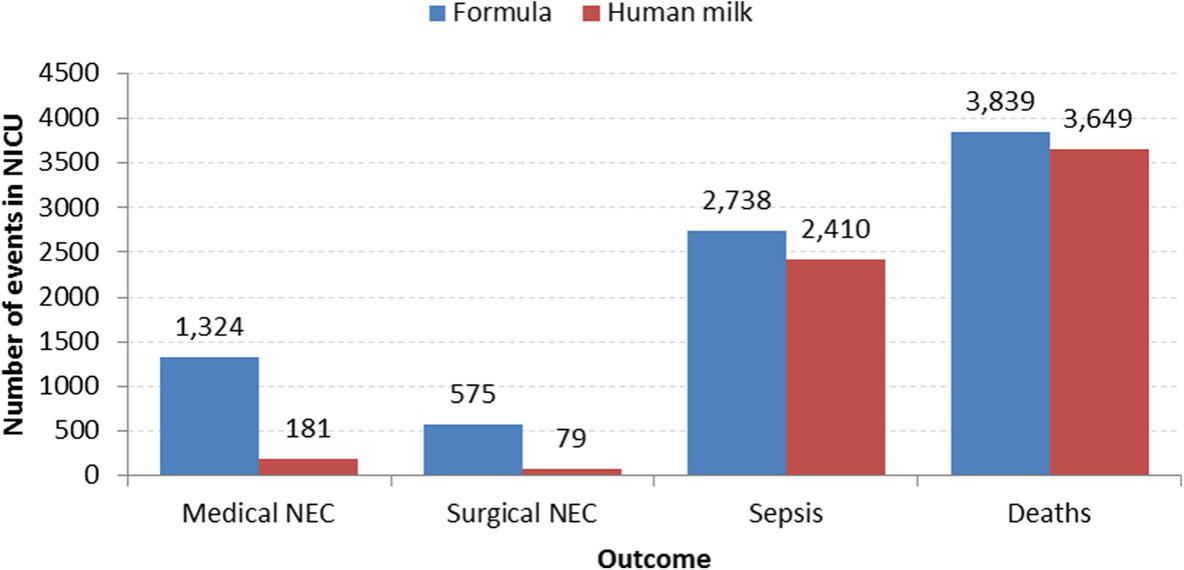
Outcomes in the NICU. Presents the expected number of events experienced by infants while in a NICU. The *red* bar represents outcomes for infants fed with mother’s own milk. The *blue* bar represents outcomes for infants fed with formula. Results are based on a population of 51,703 preterm infants. Abbreviations. NICU: neonatal intensive care unit. NEC: necrotizing enterocolitis

The estimated impact of providing human milk on outcomes in the NICU is presented in Fig. 2.

The total cost savings to the NHS due to these events is therefore estimated to be **£30.1 million** for the year. The average saving per infant would be £583.

Due to the number of deaths averted, the total QALY gain in the NICU attributed to increased levels of breastfeeding is 4,568. This is an average QALY gain per infant of 0.088. The National Institute for Health and Care Excellence (NICE) recommends a willingness to pay per QALY of £20,000 [16] Given this value, the value of the QALY gains for the cohort is equivalent to £91.4 million, and £1,767 per infant. When combined with the hospital cost savings due to fewer episodes of infection, the net value is £121.5 million.

#### Outcomes after NICU discharge

The long-term benefits associated with increased levels of breastfeeding are presented in Table 2. Disability arising from NDI was associated with the greatest impact on cost savings and QALY gain, since it is the condition that will have the longest impact over the infant’s lifetime. Reducing the rate of SIDS is also associated with a significant QALY gain. The QALY gain for leukaemia, diabetes and CHD are relatively small. This is due to diabetes and CHD occurring in the future of the infant, where outcomes will be most heavily discounted. The analysis only includes the case of these two conditions that can be attributed to obesity in childhood-other members of the cohort may develop diabetes or CHD but this will be due to risk factors other than childhood obesity. Leukaemia is a relatively rare condition, so the overall impact of reducing levels of this condition will be minimal. It is also now associated with a more optimistic outlook, with a high survival rate (and therefore a smaller QALY loss per case).

**Table 2:** Long-term benefits of increased levels of breastfeeding

Condition	Cases averted	Total cost savings	Total QALY gain
SIDS	48.03	£3,458	1,133.39
AOM	97.38	£4,480	-
Leukaemia	2.02	£231,148	3.06
NDI	259.17	£16,178,047	1,358.38
Diabetes	74.66	£121,506	11.27
CHD	46.86	£75,219	0.75

Presents the long-term benefits to the population of providing mother’s own milk to preterm infants. Results are based on a population of 51,703 preterm infants. Abbreviations: *QALY* quality-adjusted life year, *SIDS* sudden infant death syndrome, *AOM* acute otitis media, *NDI* neurodevelopmental impairment, *CHD* coronary heart disease

After discharge from NICU, total cost savings of approximately £16.6 million and a total of 6,026 additional QALYs for the cohort (£321 savings and 0.12 gained QALYs per infant) over a lifetime were estimated to be gained due to increased levels of human milk usage and exclusive breastfeeding at discharge from the NICU. Given a willingness to pay per QALY of £20,000, the value of these QALY gains for the cohort is equivalent to £120.5 million, and when combined with the cost savings, the net value is £137.1 million.

### Impact to society

The primary outcomes of this analysis are those that are relevant to the NHS or the hospital (in terms of cost savings), and to the patient (for improvement in quality of life and reduction in risk of disease and illness). There are further benefits that can be observed from a societal perspective regarding productivity gains due to averting the loss of earnings due to early death.

The analysis estimates that with the introduction of exclusive breastfeeding, there would be 190 fewer deaths due to neonatal infections and 48 fewer deaths due to SIDS. The resulting reduction in loss of lifetime economic productivity can be estimated using the estimated lifetime earnings [18]. The average lifetime productivity has been estimated at approximately £645,500 (ranging from £540,500 for low, up to £750,500 for highly educated workers). Based on 238 lives saved, a reduction of approximately £153.4 million in lifetime productivity is observed in the analysis.

### Sensitivity analysis

Deterministic sensitivity analysis (DSA) was performed to examine the effect of changes in key model parameters, where each parameter was varied according to the measure of dispersion (95% confidence intervals). Results of the DSA are presented in the format of the impact on the total cost savings for the cohort.

**Fig. 3:**
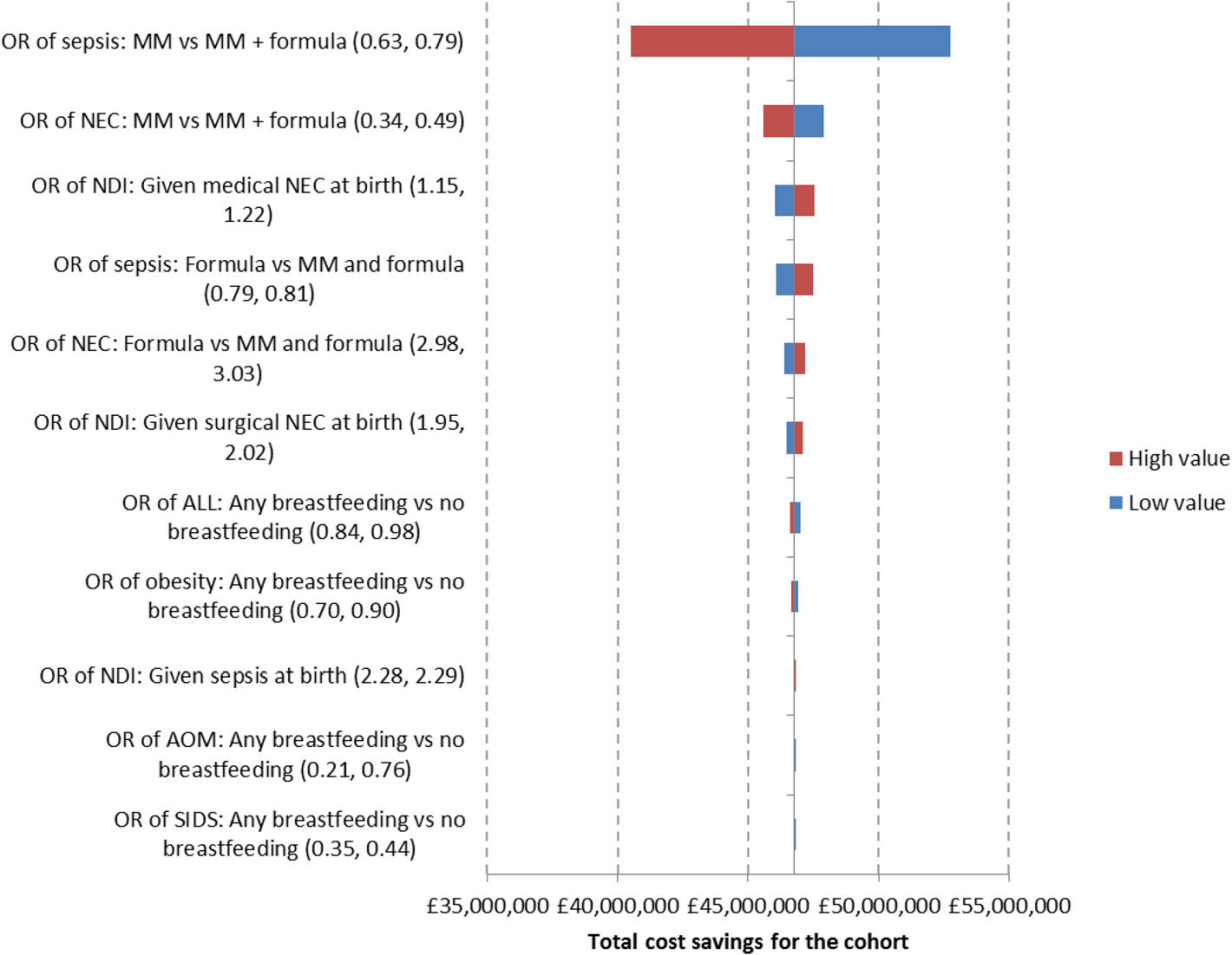
Tornado diagram for breast milk versus formula. Presents the impact of key parameters in the analysis on the potential total cost savings for the cohort of the use of mother’s milk versus formula. The *blue* bars correspond to the high value of the parameter; the *red* bars correspond to the low value of the parameter. Results are based on a population of 51,703 preterm infants. Abbreviations. OR: odds ratio. MM: mother’s milk. NDI: neurodevelopmental impairment. NEC: necrotizing enterocolitis. ALL: acute lymphoblastic leukaemia. AOM: acute otitis media. SIDS: sudden infant death syndrome

Figure 3 presents the results of the DSA. The tornado diagram indicates that results were robust to a wide range of inputs, with the odds ratio for developing sepsis and for developing NEC the variables with the largest effect on the results.

## Discussion

### Main findings

The economic model estimated that increased levels of human milk usage and breastfeeding in the NICU could save the NHS £30.1 million (£583 per infant) over the course of a year from fewer episodes of NEC and sepsis and 190 fewer deaths. The total discounted lifetime savings to the NHS for premature infants born each year from increased levels of human milk consumption in the NICU would be £46.7 million.. The average lifetime saving per infant is estimated to be approximately £904 with an average QALY gain of 0.2. This compares to a potential cost saving from a previous review of interventions to increase breastfeeding rates in neonatal units of between £66 and £586 per infant which whilst potentially lower than found in this study represents all low weight babies and not just those that were preterm [13]. Whilst the proximity of the numbers between the previous review and this study are supportive of the findings in both, the studies are of slightly different populations and this study looked at lifetime savings and QALY gains rather than the previous review that just estimated short-term savings to the NICU. In addition the previous review looked at the intervention being an activity to increase breastfeeding whilst this study looked at the maximum benefit that could be achieved from human milk as the intervention itself.

The analysis estimates that with the introduction of exclusive breastfeeding, there would be 190 fewer deaths due to neonatal infections and 48 fewer deaths due to SIDS. This is associated with an economic impact of £153.4 million in lifetime productivity.

Longer-term health benefits of human milk usage and breastfeeding in the NICU after discharge may include lower incidence of SIDS within the first year of life, otitis media, leukaemia, diabetes, CHD and disability arising from NDI. Disability was associated with the greatest impact on cost savings and QALY gain, since it is the condition that has the longest impact over the infant’s future life. The long-term outcome results were based on the calculated population of preterm infants who were able to receive some level of (as opposed to exclusive) human milk. However, it must be stated that these numbers are estimates as most evidence on outcomes identified was for term infants with no reviews providing evidence of long-term outcomes of either human milk or breastfeeding to preterm infants. Although this approach is most likely to provide conservative outcomes given that preterm infants are associated with greater rates of neurological impairment [19].

The literature review performed as a part of this research highlighted the need for more high-quality studies on outcomes of human milk and breastfeeding of preterm infants. In addition, more research is needed on the medium and long-term outcomes of breastfeeding infants. As is the case with all economic models based on the limited data that is currently available, the results should be interpreted with caution. Although, the outcomes of this study agree in principle with previous economic impact analyses [10] on increasing breast feeding rates in all babies, suggesting that the beneficial effects of human milk on the infant over the short- and long-term are real. Sensitivity analysis performed as part of this study also showed that the savings and patient benefits exist across the range of potential values that exist for breast milk benefit identified in the literature. Thus the cautionary not on whether the benefits and economic impact actually exists, but rather in relation to the strength of those benefits and the magnitude of the positive economic impact.

The results presented in this research illustrate a best-case scenario for human milk feeding for preterm infants. In reality, human milk usage and breastfeeding in the NICU may be difficult due to the infant’s condition, or mother’s own milk availability. However, increased feeding of human milk has many potential health and economic benefits to both term and preterm infants, so feeding preterm infants mother’s own breastmilk, or donor milk should be encouraged whenever possible [20]. The results presented may underestimate the true benefits of human milk usage in the long term; the results post-discharge are based on infants receiving mixed feeding (formula and human milk) up to the age of 6 months, representing a conservative scenario. There is an increase in benefit of providing human milk for longer, and a further benefit for a longer duration of exclusive breastfeeding [1]. In addition, there are also established benefits to the mother in terms of a reduced risk of breast cancer, but this has not been addressed in this analysis.

## Conclusion

The health benefits of providing human milk to preterm infants have long been established. This new analysis established that increasing human milk usage and breastfeeding rates in NICUs in England and Wales could also lead to cost savings to the NHS, relating both to the short-term reduction in infections in the NICU, and to the impact of reducing long-term associated conditions. It is important to note that no attempt was made to identify potential methods to increase human milk feeding in neonatal units for preterm infants, but the cost saving to the NHS per infant suggests that significant resource could be dedicated on interventions to raise human milk feeding rates and the intervention still be cost saving overall. The economic benefits are established and real and could potentially be realized with little effort but just a change in approach in NICUs and appropriate support to parents. More research is needed on the health and economic outcomes associated with breastfeeding, and the differences between mother’s own and donor breast milk.
